# Powdered tart cherry supplementation moderates post-exercise immunosuppression, total cholesterol, and antioxidant status with no effect on performance recovery following an acute bout of intense lower body resistance exercise

**DOI:** 10.1186/1550-2783-11-S1-P32

**Published:** 2014-12-01

**Authors:** R Dalton, K Levers, E Galvan, C Goodenough, A O’Connor, S Simbo, N Barringer, J Carter, C Seesselberg, YP Jung, A Coletta, S Mertens-Talcott, C Rasmussen, M Greenwood, R Kreider

**Affiliations:** 1 Texas A&M University, College Station, Texas, USA

## Background

Consumption of tart cherry juice has been reported to effectively reduce inflammation, muscle damage, and muscle soreness following bouts of exercise. The purpose of this study was to determine if consumption of a powdered form of tart cherries derived from tart cherry skins prior to and following intense resistance exercise promotes similar positive results as seen with tart cherry juice consumption.

## Methods

23 resistance trained men (20.9±2.6 yr, 14.2±5.4% body fat, 63.9±8.6 kg FFM) volunteered to participate in this study and were matched based on relative maximal back squat strength, age, body weight, and fat free mass. Subjects were randomly assigned to ingest, in a double blind manner, capsules containing a placebo (P, n=12) or powdered tart cherries (CherryPURE® Freeze Dried Tart Cherry Powder [TC, n=11]). Participants ingested the supplements one time daily (480 mg/d) for 10-d including day of exercise up to 48-hr post-exercise. Participants performed 10 sets of 10 repetitions at 70% of 1RM barbell back squat with 3 minutes recovery between sets, maintaining equivalent average work values between groups throughout the protocol (p=0.24). Isokinetic knee extension/flexion maximal voluntary contractions (MVCs) and fasting blood samples were taken pre-squat workout, 60-minutes following the squat workout as well as after 24 and 48 hours of recovery and analyzed by MANOVA with repeated measures. Consent to publish the results was obtained from all participants.

## Results

Overall changes in WBC, LYMPH (p<0.001), extension/flexion MVCs (p<0.001), and RBC, HCT, TG, TotCHL (p=0.048) were observed in both groups over time, but the overall Wilks’ Lambda MANOVA analysis did not reveal a significant group x time effect for WBC and LYMPH (p=0.39); extension/flexion MVCs (p=0.83); RBC, HCT, TG, and TotCHL (p=0.42). MANOVA univariate analysis revealed significant effects in LYMPH (p<0.001) in both groups over time, but no significant effects were observed over time in WBC (p=0.151). MANOVA univariate analysis revealed a significant group x time linear effect was shown for LYMPH (p=0.013) in addition to a trend toward a significant delta value based on group assignment for LYMPH (p=0.10). An overall trend for SOD and TAS changes in both groups over time (p=0.076) was demonstrated, but the overall MANOVA analysis did not reveal a significant group x time effect (p = 0.29). Further, a group x time cubic effect for SOD (p=0.046) was indicated in the univariate analysis. MANOVA uni-variate analysis indicated a significant group x time linear effect for TotCHL (p=0.009) with a significant delta value based on group assignment (p=0.014). No significant group x time effects were found for RBC (p=0.97), HCT (p=0.76), and TG (p=0.46). MANOVA univariate analysis revealed significant effects for all EXT MVCs [I-III] (p<0.001) in both groups over time, but no significant group x time effects were found for all FLEX MVCs [I] (p=0.25), [II] (p=0.21), [III] (p=0.46).

## Conclusion

Results of this study indicate that short-term supplementation with powdered tart cherries over the 7 days leading up to, during, and 2 days after intense resistance exercise aids in overcoming the post-exercise immunosuppression (PEIS) phenomenon typically seen in trained athletes as indicated by a significantly greater post-exercise LYMPH response compared to the placebo. Further, as a result of powdered tart cherry supplementation compared to a placebo, the SOD response was also significantly lower suggesting a diminished release of ROS in response to the resistance exercise bout. Performance recovery as assessed from isokinetic knee extension and flexion exercise was unaffected by supplementation with powdered tart cherry versus a placebo. Overall, these findings suggest that supplementation with a powdered tart cherry product surrounding an intense resistance event reduces the effect of PEIS in trained individuals, total serum cholesterol levels, and the free radical response typically associated with intense resistance exercise. Further research is necessary to determine long-term supplementation effects with resistance training.

